# PD-L1^+^ neutrophils mediate immune regulation of CD8^+^ T cells in halo nevi

**DOI:** 10.3389/fimmu.2025.1628913

**Published:** 2025-08-20

**Authors:** Yixuan Zhang, Yingying Xu, Wenjun Cui, Haoyang Wang, Min Li, Lu Liu

**Affiliations:** ^1^ Department of Dermatology, The Fourth Affiliated Hospital of Soochow University (Suzhou Dushu Lake Hospital, Medical Center of Soochow University), Suzhou, China; ^2^ Department of Burns and Plastic Surgery, Affiliated Suzhou Hospital of Nanjing Medical University, Suzhou, China

**Keywords:** immunomodulatory therapy, halo nevi, neutrophils, PD-L1, melanocytes

## Abstract

**Background:**

Halo nevi are clinically common and are characterized by a circle of leukoderma around the central melanocytic nevus. Studies have shown that the pathogenesis of halo nevi is similar to that of vitiligo and is associated with the role of CD8⁺ T lymphocytes in melanocyte destruction. Histopathological findings have revealed neutrophil infiltration in halo nevi; however, the specific immune mechanisms involving neutrophils have not been thoroughly investigated. In the present study, we investigated the role of neutrophils in halo nevi using histopathological and immunological analyses.

**Methods:**

To this end, we examined the infiltration patterns of immune cells in halo nevi, with a particular focus on IFN-γ-induced PD-L1 expression in neutrophils and its potential immunoregulatory effects.

**Results:**

The results demonstrated that IFN-γ expression in the lesional skin of halo nevi contributed to the induction of PD-L1 expression in neutrophils. PD-L1⁺ neutrophils promoted apoptosis and suppressed the function of CD8⁺ T lymphocytes. Notably, some halo nevi showed a tendency to spontaneous regression, but the underlying mechanisms remain unclear, and this regulatory mechanism influences the local immune response and may facilitate the repigmentation of the surrounding leukoderma in halo nevi.

**Conclusions:**

This study is the first to explore the involvement of neutrophils in halo nevi and reveal the potential immunoregulatory role of PD-L1 in this process. The elucidation of this mechanism not only provides a more comprehensive understanding of autoimmune skin diseases but may also offer new strategies for targeted therapy in other related disorders, such as vitiligo.

## 1 Introduction

Halo nevi are relatively common, occurring in approximately 1% of the population (1–4). They are characterized by a depigmented ring surrounding a central melanocytic nevus and are frequently associated with vitiligo, with the two conditions potentially occurring either sequentially or concurrently (4). Based on this association, some researchers have proposed that halo nevi represent a special subtype of vitiligo (3, 5), which is further supported by the evidence that CD8^+^ T lymphocytes contribute to melanocyte destruction in both conditions (3, 6, 7). Generally, halo nevus tissues are more readily accessible than vitiligo tissues. Therefore, researchers have considered studying autoimmune signaling in halo nevus skin samples as an alternative strategy for vitiligo research (3).

Some patients with halo nevi exhibit spontaneous regression of both the central nevus and the surrounding leukoderma. This process typically progresses through four stages: stage 1, appearance of a depigmented macule surrounding the central nevus; stage 2, gradual fading of the central nevus; stage 3, complete disappearance of the central nevus, leaving only the depigmented area; stage 4, partial or complete repigmentation of the depigmented area (1). Complete regression of halo nevi may take several months to years (1, 8, 9), and it has been reported that, eventually, the central nevus disappears completely in at least 50% of patients (10). The regression of the central nevus is associated with the activation of CD8^+^ T lymphocytes targeting melanocytes (11), whereas the mechanism underlying the regression of the surrounding leukoderma remains unclear (1). As nevus cells disappear and repigmentation of leukoderma occurs, T lymphocytes also gradually decrease (12, 13); however, no in-depth research has focused on the reduction of T lymphocytes and the repigmentation of leukoderma in halo nevi. This is an important issue because patients with vitiligo may benefit from studying this analogous condition to uncover new therapeutic opportunities.

Additionally, histopathological examination of the halo nevi in a past study revealed neutrophil infiltration (13), suggesting that the development of halo nevi may be associated with a local immune response involving neutrophils. However, the specific mechanisms underlying this process have not been thoroughly investigated. Therefore, we aimed to further explore the role and mechanisms of neutrophils in halo nevi. It has been demonstrated that IFN-γ can be produced by CD8^+^ T cells and is expressed in the lesional skin of both vitiligo and halo nevi (14, 15). Additionally, IFN-γ has been shown to induce the expression of PD-L1 in various cell types (16). Based on these findings, we hypothesized that IFN-γ may induce PD-L1 expression in neutrophils within halo nevi, thereby modulating the local immune response.

In this study, we found that neutrophils in halo nevi tissues express PD-L1 and characterized the unique biological properties of PD-L1^+^ neutrophils, including chemotaxis, reactive oxygen species (ROS) generation, phagocytosis, degranulation, and apoptosis through a comprehensive functional analysis. Finally, we demonstrated the immune regulatory effect of PD-L1^+^ neutrophils on CD8^+^ T cells, showing that PD-L1^+^ neutrophils promote CD8^+^ T cell apoptosis and suppress their functional capacity. This immunosuppressive effect is mediated through PD-L1, as the blockade of PD-L1 restores CD8^+^ T cell survival and cytokine production. Since the appearance of leukoderma is attributed to CD8^+^ T cell-mediated cytotoxicity in halo nevi (3), the mechanism we verified may contribute to the repigmentation process. Our findings provide insights into the role and mechanisms of neutrophils in halo nevi, and we hope to offer new perspectives that may serve as a reference for the treatment of both halo nevi and vitiligo.

## 2 Materials and methods

### 2.1 Ethics approval and consent

This study was approved by the Medical Ethics Committee of the Fourth Affiliated Hospital of Soochow University. Skin samples from patients with halo nevus were collected after informed consent was obtained from all participants. Blood sample collection from healthy donors was approved by the Medical Ethics Committee of Affiliated Suzhou Hospital of Nanjing Medical University, and written informed consent was obtained from all patients. All procedures were conducted in accordance with the institutional guidelines and ethical standards.

### 2.2 Clinical patients and samples collection

From July 2022 to October 2023, six patients diagnosed with halo nevi who underwent lesion excision at the Department of Dermatology, Fourth Affiliated Hospital of Soochow University, were enrolled in this study. The participants were four female and two male patients, aged 16 to 38 years (mean age: 24.25 ± 6.18 years). Discarded skin tissues were collected postoperatively, fixed in formalin at room temperature (RT), and stored for subsequent analysis. Inclusion criteria were as follows: 1) clinical diagnosis of halo nevus; 2) age ≥ 12 years; 3) willingness to undergo surgical excision of the lesion; 4) provision of signed informed consent. The exclusion criteria were as follows: 1) pregnancy or lactation; 2) keloid tendency, bleeding disorders, active infections, or severe dysfunction of the heart, liver, or kidneys; 3) coexisting hematologic or malignant diseases; and 4) refusal to provide informed consent.

Peripheral blood samples were collected from six healthy volunteers (three females and three males, mean age: 24.5 ± 8.36 years) between October 2022 and May 2023 at the Affiliated Suzhou Hospital of Nanjing Medical University. These donors underwent routine physical examinations and were not diagnosed with skin diseases. Each volunteer provided 10 mL of peripheral blood via elbow venipuncture. Samples were kept at 4°C and processed within 2 h to isolate neutrophils and CD8^+^ T cells. Inclusion criteria for healthy volunteers were: 1) no known dermatologic conditions; 2) age ≥ 12 years; 3) normal blood count, liver and kidney function, electrolytes, and coagulation profiles; 4) written informed consent. The exclusion criteria were as follows: 1) history of chronic systemic diseases; 2) use of vasoactive medications within the past six months; 3) acute infections within the past month; and 4) failure to provide informed consent.

### 2.3 Isolation of neutrophils and CD8^+^ T cells

Neutrophils and CD8^+^ T cells were purified within 2 h of blood collection. According to the manufacturer’s protocols, cells were extracted using the EasySep™ Direct Human CD8^+^ T Cell Isolation Kit (STEMCELL Technologies) and the Direct Human Neutrophil Isolation Kit (STEMCELL Technologies). Neutrophils and CD8^+^ T cells were suspended in RPMI-1640 (Gibco) supplemented with 10% fetal bovine serum (Gibco) for maintenance.

### 2.4 Cell stimulation

Neutrophils were stimulated with IFN-γ at a concentration of 10 ng/mL (Sigma) for 24 h. After stimulation, PD-L1^+^ and PD-L1^-^ neutrophils were sorted by flow cytometry and then co-cultured with CD8^+^ T cells from the same volunteer at a 2:1 ratio for 12 h. For the PD-L1 blockade experiment, IFN-γ-treated neutrophils, PD-L1 monoclonal antibody Atezolizumab (TECENTRIQ^®^, 10 μg/mL), and IFN-γ-treated neutrophils were co-cultured with CD8^+^ T cells for 12 h, followed by other assessments.

### 2.5 Immunofluorescence and image analysis

Formalin-fixed, paraffin-embedded tissue samples from halo nevi patients were sectioned at a thickness of 5 μm. Paraffin sections were deparaffinized using xylene and a graded ethanol series. After incubation with 3% hydrogen peroxide (Sinopharm Chemical Reagent Co.) at RT for 10 min, antigen retrieval was performed using 1 mM Tris-EDTA (pH = 9.0). The sections were blocked with 5% bovine serum albumin (BSA, Solarbio) at RT for 20 min, and the primary antibody was added and incubated at RT for 30 min. A Bond Polymer Refine Detection Kit (Leica, Wetzlar, Germany) was used according to the manufacturer’s protocol and incubated at RT for 10 min. The sections were then incubated with the corresponding Neon TSA fluorophores (1:100, Shanghai Yuanxi Biotechnology Co.) at RT for 10 min. After each staining cycle, the sections were subjected to antigen retrieval, and the process was repeated for sequential antibody staining. Between each step, the sections were washed three times with PBS. Nuclei were stained with DAPI and incubated at RT in the dark for 10 min. Images were acquired using a fluorescence microscope (Nikon). The primary antibodies used were anti-PD-1 (1:400; Sino Biological), anti-CD8 (1:300; Bioss), anti-PD-L1 (1:400; CST), anti-CD66B (1:3000; Abcam).

The stained tissue slides were scanned using the Orion imaging system (RareCyte). The acquired images were imported into the HALO digital pathology platform for analysis. HALO AI performed nucleus segmentation-based cell identification for each sample across all specimens. Subsequently, HALO AI was used for tissue classification to identify PD-L1^+^ neutrophil and CD8^+^ T cell regions within the entire tissue area. These algorithms were embedded in the HALO Spatial Plot module, enabling the spatial distribution analysis of PD-L1^+^ neutrophils and CD8^+^ T cells based on cell phenotyping.

Human peripheral blood neutrophils were isolated and washed twice with PBS. The cells were fixed with 4% paraformaldehyde at RT for 15 min, followed by permeabilization with 0.01% Triton X-100 (Solarbio) for 30 min. After blocking with 5% BSA at RT for 1 h, the cells were incubated with primary antibodies, CD35 (1:200, Abcam) and CD63 (1:200, Abcam), at 4°C overnight. The following day, secondary antibodies, Alexa Fluor 488 IgG (1:500, Invitrogen) and CY3 IgG (1:500, Jackson ImmunoResearch), were added and incubated at RT for 1 h. PBS washes (three times) were performed between each step. Images were acquired using a confocal microscope (ZEISS).

### 2.6 Transcriptome sequencing of IFN-γ-stimulated neutrophils

Neutrophils were collected from both the IFN-γ-stimulated (experimental) group and the unstimulated (control) group. Total RNA was extracted using the TRIzol reagent (Invitrogen) according to the manufacturer’s protocol. RNA quantity was assessed using Qubit 4.0 (Invitrogen), and RNA quality was examined by denaturing agarose gel electrophoresis. RNA libraries were prepared using the VVAHTS^®^ Universal V8 RNA-seq Library Prep Kit for Illumina (NR605-0, Vazyme) and sequenced on the Illumina NovaSeq 6000 platform with a 150-bp paired-end sequencing strategy. The mRNA enrichment, library construction, sequencing, and data analysis were performed using the Yunbios platform. Raw sequencing data were processed using Skewer (v0.2.2) and the quality was assessed using FastQC (v0.11.2). The sequencing reads were 2×150 bp long. Read indexing and mapping to the human reference genome (GRCh38/hg38) was performed using Hisat2 (v2.0.0), followed by transcriptome assembly using StringTie (v2.1.4). Gene expression levels were quantified as Fragments per kilobase of transcripts per million mapped reads (FPKM). Principal component analysis was performed on all samples and their corresponding gene expression levels.

### 2.7 Bioinformatics for RNA sequencing data

Differentially expressed genes (DEGs) between the control group and the IFN-γ-treated group were identified using DESeq2 software (v1.16.1) based on the FPKM values of different transcripts. The thresholds for determining DEGs are *P* < 0.05 and an absolute fold change ≥ 2. The following analyses were performed using OmicShare, a free online platform for data analysis (https://www.omicshare.com/tools). Hierarchical clustering analysis was performed on the DEGs between the control and IFN-γ groups. The data were normalized, and the Euclidean distance was used to calculate the distance between data points. Complete linkage was applied to determine the similarity during clustering, and the results were visualized as a heatmap. A volcano plot was generated with the log_2_(FC) on the x-axis and -log_10_(p) on the y-axis. Differentially significant genes are highlighted in distinct colors to visually represent the significance of differences between the two sample groups. We performed Gene Ontology (GO) annotation and Kyoto Encyclopedia of Genes and Genomes (KEGG) pathway enrichment analysis for the DEGs. GO enrichment analysis was performed using TopGO software (https://www.bioconductor.org/packages/release/bioc/html/topGO.html). KEGG pathway analysis was performed based on the KEGG database (https://www.genome.jp/kegg/pathway.html) for functional annotation of gene pathways, with significantly enriched pathways selected using a threshold of *P* < 0.05.

### 2.8 Flow cytometry

Single-cell suspensions with high viability were obtained from skin tissues using a tissue dissociation kit (Miltenyi Biotec) in combination with a fully automated tissue processor (Cytiva), yielding a final concentration of 5 × 10^6^ cells/mL. The flow cytometry setup included blank controls, compensation controls, and sample tubes. Fluorescently labeled surface antibodies (CD66B, CD274, CD182, CD80, and HLA-DR) (Shanghai Universal Biotech Co., Ltd.) were added to the sample tubes following the manufacturer’s instructions and incubated at 4°C in the dark for 30 min. The cells were then washed with 1 mL PBS, centrifuged at 300 g for 5 min, and resuspended in 400 µL PBS for flow cytometry analysis (BD Biosciences) within 3 h. For apoptosis detection, antibodies and propidium iodide (PI) (BD Biosciences) were added according to the manufacturer’s instructions. The samples were incubated in the dark at RT for 15 min before immediate analysis. For ROS detection in neutrophils, cells were stimulated with 10 nM PMA (phorbol 12-myristate 13-acetate; Solarbio) for 30 min, washed with PBS, and directly incubated with 10 mM H_2_DCFDA (2’,7’-dichlorodihydrofluorescein diacetate) fluorescent probe (Solarbio) at 37°C for 30 min. Neutrophil phagocytosis was assessed using the pHrodo™ E. coli BioParticles Phagocytosis Kit (Thermo Fisher Scientific). Neutrophils were incubated with pHrodo™ E. coli particles at 37°C under 5% CO_2_ conditions for 30 min. Phagocytic capacity was evaluated by measuring the fluorescence intensity of internalized particles. Flow cytometry data were processed using the FlowSOM plugin or FlowJo v10.

### 2.9 Agarose neutrophil chemotaxis model

An agarose-based neutrophil chemotaxis model was established as previously described (17). A 1.2% agarose solution was mixed with a culture medium containing 50% Hanks’ balanced salt solution (HBSS) with Ca²^+^ and Mg²^+^ and 50% RPMI 1640 (with 20% heat-inactivated fetal bovine serum) at a 1:3 ratio, and then poured into a 35 mm culture dish to solidify. Three wells with a diameter of 3 mm and spacing of 2.8 mm were cut into the gel. The central well was filled with the chemoattractant N-formyl-Met-Leu-Phe (fMLP, 0.1 µM), while the side wells were filled with a neutrophil suspension at a concentration of 1×10^7^ cells/mL. Samples were incubated at 37°C with 5% CO_2_ for 2 h, followed by microscopic observation (Olympus IX71) to record neutrophil chemotactic distance and migration patterns. The images were analyzed using a Cellular Chemotaxis Analysis Platform (CCAP).

### 2.10 Cytokine detection

Single-cell cytokine profiling of CD8^+^ T cells was performed using an IsoCode Chip (Isoplexis USA). Approximately 30 μL of cell suspension was loaded onto the chip and incubated at 37°C under 5% CO_2_ conditions for 16 h. The chip utilizes 32-plex antibody barcode technology to capture protein secretions from 400 to 1000 single cells, followed by fluorescent ELISA-based detection and analysis (18). IsoSpeak 3.0 software was used to assess the secretion profiles of 32 cytokines, including IFN-γ, TNF-α, GM-CSF, IL-6, IL-10, and others.

Neutrophils pretreated with IFN-γ or co-treated with PD-L1 monoclonal antibody (Atezolizumab) and IFN-γ were co-cultured with CD8^+^ T cells for 4 h. CD8^+^ T cells were subsequently sorted, and cytokine levels, including IL-2, IL-4, IL-6, IFN-γ, TNF, IL-17A, and IL-10, were quantified using the Cytometric Bead Array (CBA) kit (BD Biosciences) following the manufacturer’s instructions.

### 2.11 Statistical analysis

Data are presented as mean ± standard deviation (SD). Normality was assessed using the Shapiro-Wilk test. For comparing multiple groups, one-way analysis of variance (ANOVA) was used for continuous variables with a normal distribution. The Kruskal-Wallis test was used to compare continuous variables with skewed distributions. The Bonferroni *post hoc* test was used for multiple comparisons. Student’s t-test or Wilcoxon signed-rank test was used to compare the differences between two groups. All analyses were conducted using GraphPad Prism(version 10.0; San Diego, CA, USA). *P* < 0.05 was considered statistically significant.

## 3 Results

### 3.1 Infiltration of CD8^+^ T Cells and neutrophils in halo nevi with elevated IFN-γ expression

**Figure 1:**
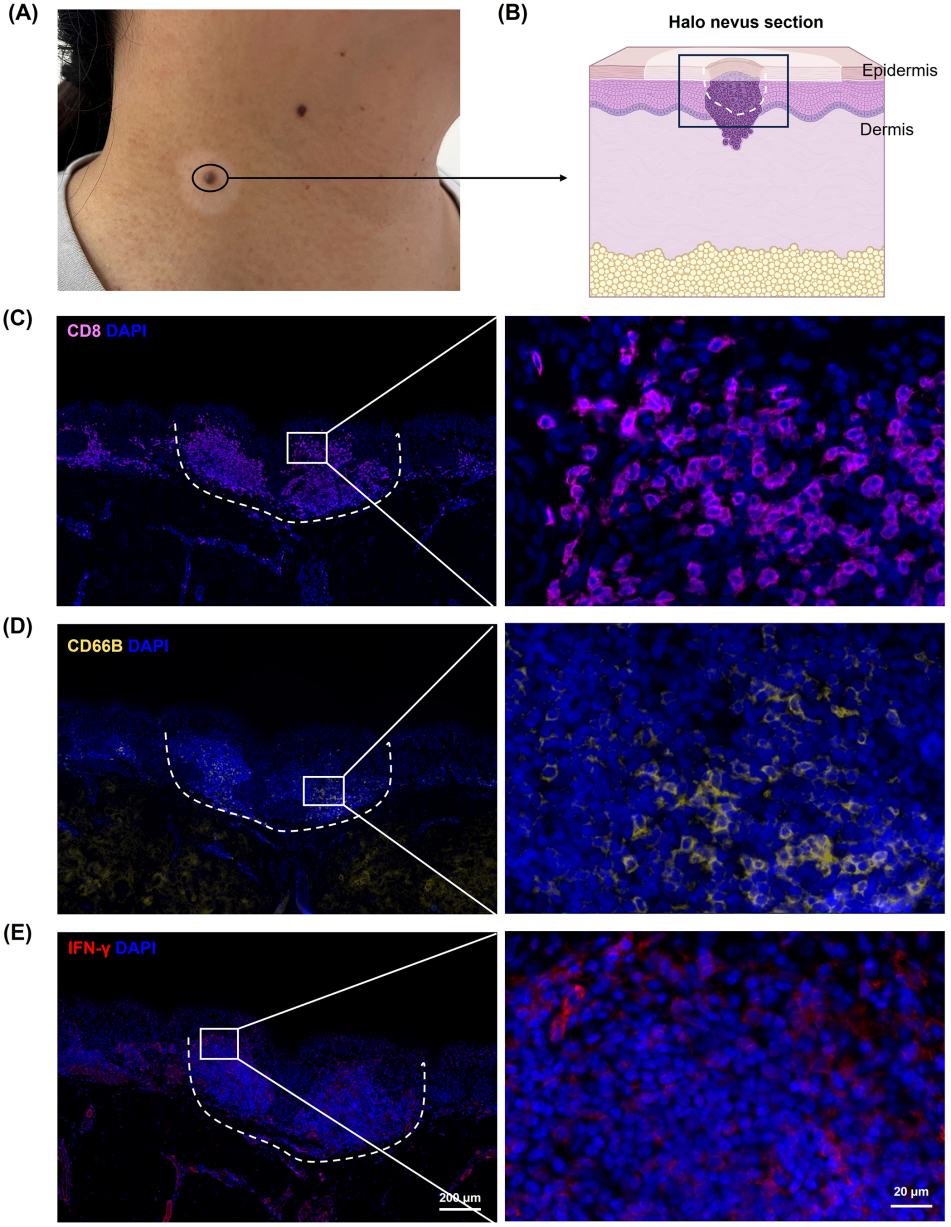
Immune microenvironment in halo nevi. **(A)** Clinical image of lesional skin from a patient with halo nevus. **(B)** Schematic diagram of a tissue section from a halo nevus. Immunofluorescence staining showing CD8^+^ T cells **(C)**, neutrophils (CD66B) **(D)**, and IFN-γ protein expression **(E)** in halo nevi tissues. The dotted line marks the central nevus area.

To investigate the characteristics of the immune microenvironment in halo nevi, skin biopsies were obtained from six patients with halo nevi (**Figures 1A, B**). Immunofluorescence staining revealed substantial infiltration of CD8^+^ T cells in both the central nevus of the halo nevi and the surrounding leukoderma area (**Figure 1C**). Meanwhile, immunofluorescence staining revealed a marked upregulation of IFN-γ in halo nevi (**Figure 1E**). In four patients, neutrophils (CD66B^+^) were widely distributed in both the central nevus and the surrounding leukoderma (**Figure 1D**). These results suggest that the formation of halo nevi may involve a complex immune response network, including CD8^+^ T cell-mediated cytotoxic immune attacks and immune responses involving neutrophils. Further, the elevated expression of IFN-γ indicates an active immune response within the local microenvironment (19, 20).

### 3.2 High levels of IFN-γ induced PD-L1 expression in neutrophils

**Figure 2:**
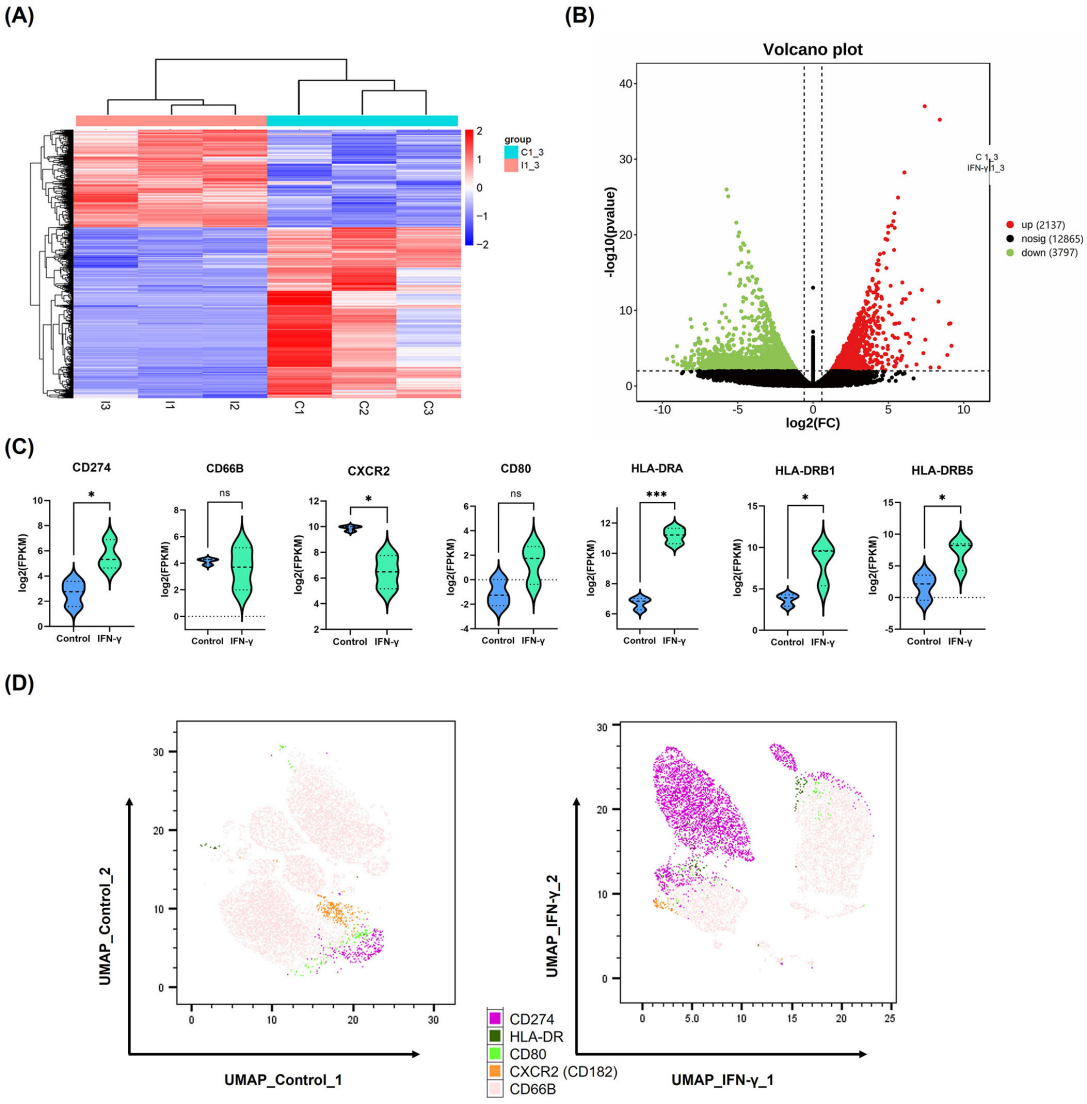
High levels of IFN-γ induce PD-L1 expression in neutrophils. **(A, B)** Heatmap and volcano plot showing differentially expressed genes between IFN-γ–stimulated (10 ng/mL) and control neutrophils based on mRNA sequencing data. **(C)** Violin plots depicting mRNA expression levels of selected immune-related genes in neutrophils after IFN-γ stimulation. **(D)** UMAP plot of multicolor flow cytometry analysis showing differential protein expression between IFN-γ–stimulated and control neutrophils. *P <.05, ***P <.001. ns, not significant. mRNA, messenger RNA; UMAP, UMAP, Uniform Manifold Approximation.

Considering the high IFN-γ levels and neutrophil infiltration observed in halo nevi, we investigated the potential impact of IFN-γ on neutrophil function. In this context, we noted that IFN-γ is widely recognized for its role in modulating the checkpoint inhibitor PD-L1 expression (15, 21). As one of the primary stimulatory factors for PD-L1 expression, IFN-γ stimulates its upregulation in various cell types (16). To simulate the high IFN-γ microenvironment characteristic of halo nevi and to validate that IFN-γ induces high PD-L1 expression in neutrophils, human neutrophils were stimulated with 10 ng/mL IFN-γ for 24 h as the IFN-γ treatment group, while neutrophils cultured without IFN-γ for 24 h served as the control group. The two groups were subjected to transcriptome sequencing and multi-color flow cytometry staining. Clustering analysis revealed a distinct difference in mRNA expression between the IFN-γ treatment group and the control group (**Figure 2A**). The differential gene expression volcano plot showed the upregulation of 2,137 genes in the IFN-γ treatment group (**Figure 2B**). Several immune-related genes were selected, and the mRNA expression of PD-L1 (CD274) was significantly increased (**Figure 2C**). In contrast, UMAP (Uniform Manifold Approximation and Projection) dimensionality reduction analysis was performed on multicolor flow cytometry data for PD-L1, HLA-DR, CD80, CXCR2 (CD182), and CD66B. The results indicated that IFN-γ significantly induces *PD-L1* gene expression in neutrophils and increases the proportion of PD-L1^+^ neutrophil subpopulations (**Figure 2D**).

Principal Component Analysis showed that the first 2 PCs account for 83.43% and 16.67% of the observed variance in the dataset, respectively, effectively distinguishing the samples based on IFN-γ stimulation (**Supplementary Figure 1A**). To investigate the biological changes in neutrophils stimulated with IFN-γ, GO and KEGG analyses were performed (**Supplementary Figures 2B, C**). The results showed that IFN-γ stimulation induced neutrophil activation (GO:0042119) and transmembrane signaling receptor activity (GO:0004888). Interestingly, GO terms related to the plasma membrane (e.g., GO:0005887, GO:0016020, and GO:0098805) were enriched, potentially reflecting changes in the expression of membrane proteins such as PD-L1. PD-L1 is a type I transmembrane protein whose functional expression depends on its membrane localization (22). Additionally, the KEGG analysis revealed activation of the MAPK signaling pathway. Previous studies have shown that the MAPK/ERK pathway is involved in the regulation of PD-L1 expression induced by IFN-γ (23). Therefore, the RNA sequencing data not only demonstrated that IFN-γ induced PD-L1 expression in neutrophils but also uncovered the biological activities and potential signaling pathways involved.

### 3.3 PD-L1 expression in neutrophils within halo nevi and their spatial proximity with CD8^+^ T cells

**Figure 3:**
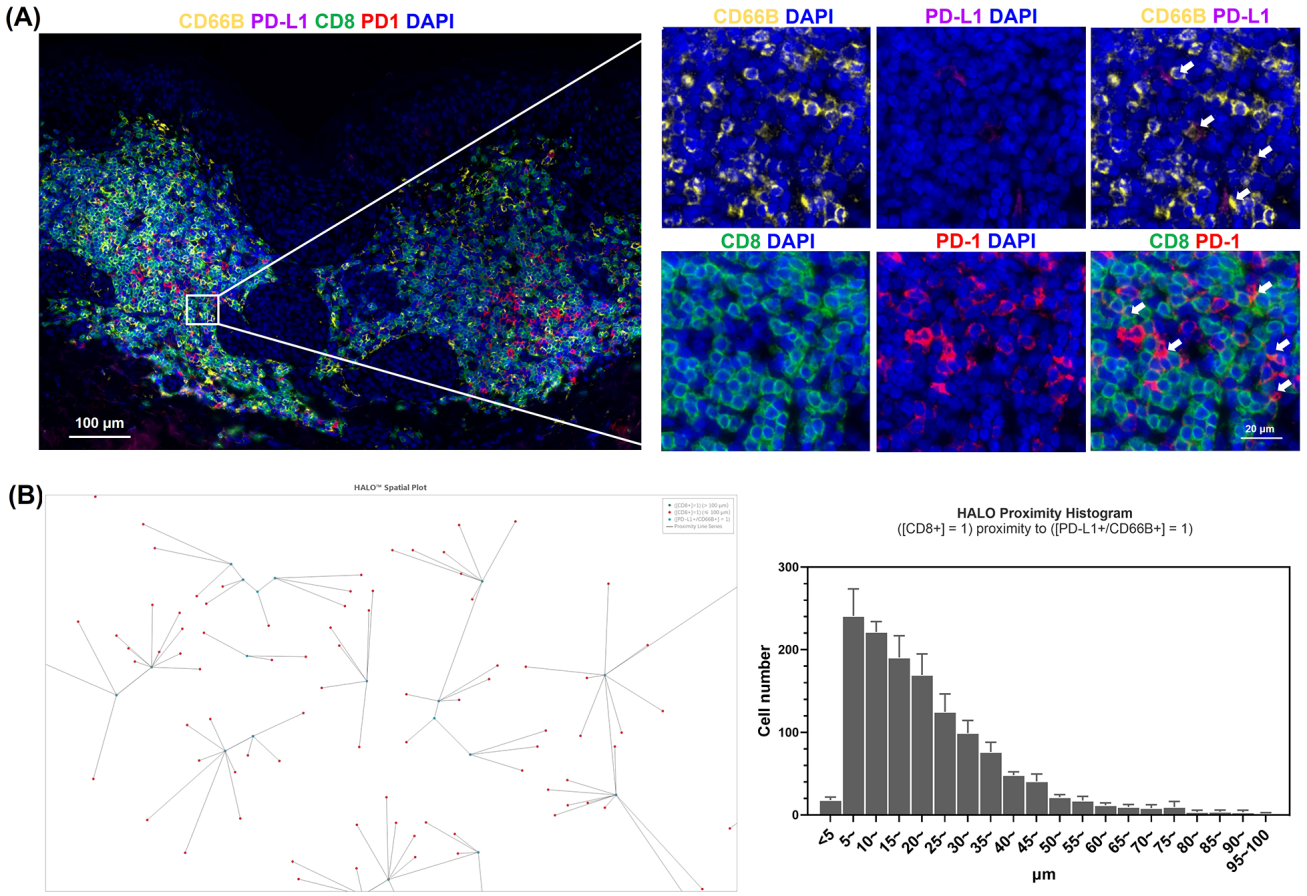
Phenotypic characterization of neutrophils in halo nevus lesions. **(A)** Immunofluorescence staining of halo nevus lesions and surrounding depigmented areas with CD8, CD66B, PD-L1, and PD-1 (the upper right white arrow indicates PD-L1^+^ neutrophils; the lower right white arrow indicates PD-1^+^/CD8^+^ T cells). **(B)** Representative image from HALO analysis showing the spatial relationship between CD8^+^ T cells and PD-L1^+^ neutrophils (right). HALO proximity histogram showing the number of CD8^+^ T cells within 100 μm of PD-L1^+^ neutrophils (left). Six randomly selected images were analyzed to quantify the distribution of CD8^+^ T cells within a 100 μm radius centered on PD-L1^+^ neutrophils.

The elevated expression of IFN-γ in halo nevi is well established, and our *in vitro* experiments confirmed that high levels of IFN-γ can induce PD-L1 expression in neutrophils. To determine whether neutrophils in halo nevi express PD-L1 *in situ*, we performed immunofluorescence staining of skin lesions from patients with halo nevi (**Figure 3A**). The results demonstrated that neutrophils (CD66B) in the halo nevi expressed PD-L1, whereas CD8^+^ T cells (CD8) expressed PD-1 on their surfaces (**Figure 3A**). To further confirm the spatial relationship between PD-L1^+^ neutrophils and CD8^+^ T cells, the imaging data were analyzed using the HALO digital pathology platform (**Figure 3B**). Six randomly selected images were scanned, with PD-L1^+^ neutrophils as the central reference point, and the distribution of CD8^+^ T cells within a 100 μm radius was quantified. HALO spatial analysis revealed that the spatial distance between PD-L1^+^ neutrophils and CD8^+^ T cells was mostly <20 μm. The proximity between CD8^+^ T cells and PD-L1^+^ neutrophils facilitates intercellular interaction, and PD-L1, which is highly expressed on neutrophils, can directly bind to PD-1 on CD8^+^ T cells, potentially modulating the cellular effector functions.

### 3.4 Functional characterization of PD-L1^+^ neutrophil subsets

**Figure 4:**
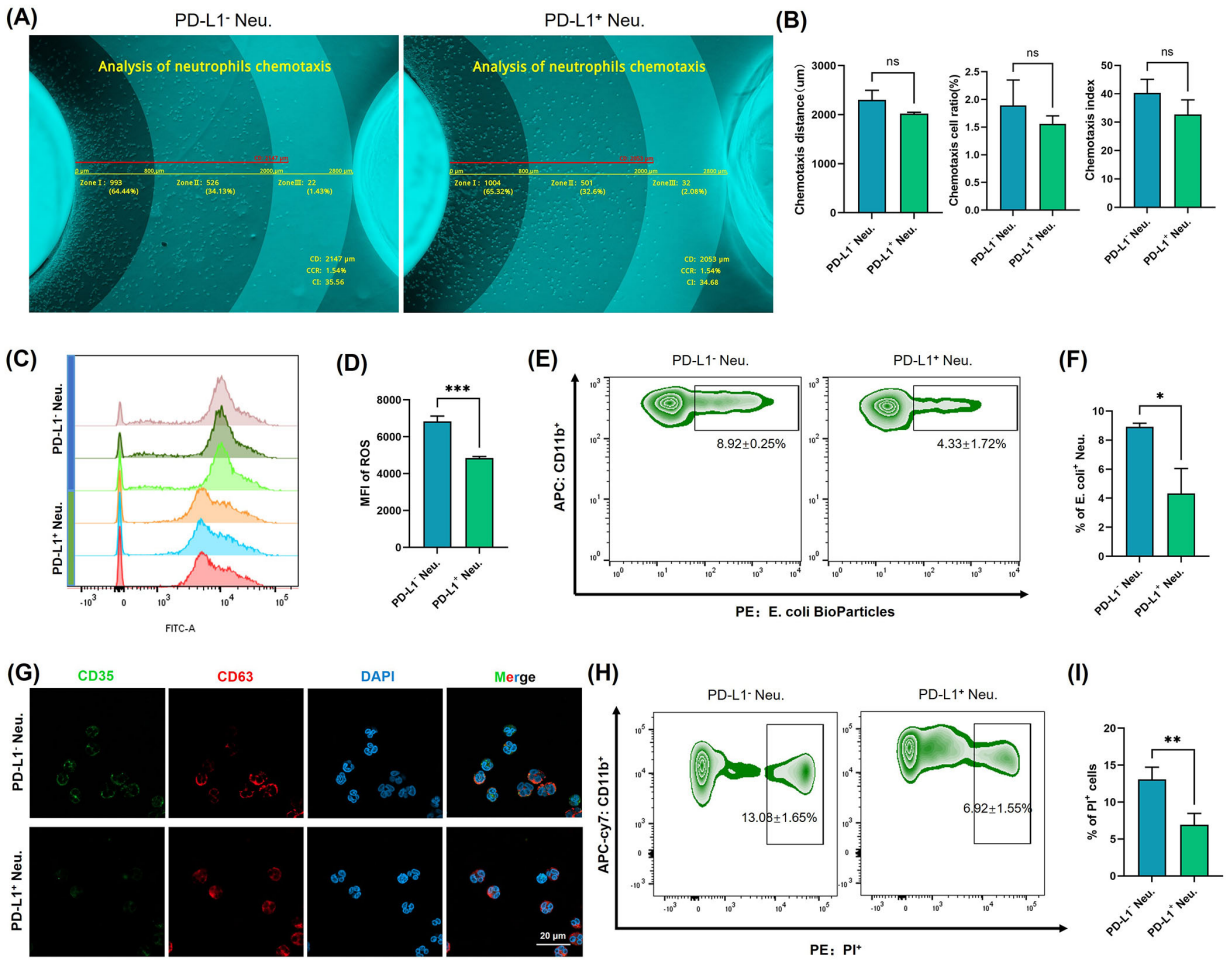
Functional characterization of PD-L1^+^ neutrophil subsets. **(A)** Neutrophil chemotaxis was assessed using a computer vision-based analysis platform in PD-L1^+^ neutrophils and PD-L1^-^ neutrophils. Representative original images were automatically measured and annotated. **(B)** Quantitative analysis of CD, CCR, and CI (n = 3). **(C)** ROS were labeled using fluorescent probes and detected by flow cytometry in PD-L1^+^ neutrophils and PD-L1^-^ neutrophils. **(D)** Quantification of ROS levels by MFI (n = 3). **(E)** Phagocytosis of neutrophils was assessed with fluorescently labeled **(E)** coli bioparticles in PD-L1^+^ neutrophils and PD-L1^-^ neutrophils. **(F)** Quantification of the rate of **(E)** coli positive normalized to unstimulated controls (n = 3). **(G)** Expression of CD35 and CD63 in PD-L1^+^ neutrophils and PD-L1^-^ neutrophils was detected by immunofluorescence staining and visualized using confocal microscopy. Bar=20 μm. **(H)** Apoptosis of different neutrophil subsets was analyzed by flow cytometry (n = 3). **(I)** Quantification of the rate of PI positive normalized to unstimulated controls. All the images are representative. *P <.05, **P <.01, ***P <.001. ns, no significant difference. CD, chemotaxis distance; CCR, chemotaxis cell ratio; CI, chemotaxis index; ROS, reactive oxygen species; MFI, mean fluorescence intensity; Neu., neutrophils, PI, propidium iodide.

PD-L1^+^ neutrophil subsets were subjected to a series of functional assays to evaluate the biological characteristics of the immune response. We isolated neutrophils from the peripheral blood of healthy volunteers and stimulated them with IFN-γ at a concentration of 10 ng/mL for 24 h. PD-L1^+^ and PD-L1^-^ neutrophil subsets were then sorted by flow cytometry and subjected to subsequent analyses. To evaluate the chemotactic capacity of neutrophils, we employed an agarose migration model coupled with a computer vision-based chemotaxis analysis platform that provided an intuitive and quantitative representation of neutrophil migration toward chemoattractant peptides. The chemotactic function was comprehensively evaluated using three parameters: chemotaxis distance (CD), chemotaxis cell ratio (CCR), and chemotaxis index (CI). The CD was further categorized into <800 μm, 800–2000 μm, and >2000 μm, allowing for a detailed zonal analysis of migration patterns. Quantitative analysis of chemotactic maps revealed no significant differences (ns) in CD, CCR, or CI between PD-L1^+^ and PD-L1^-^ neutrophils (**Figures 4A, B**). The results showed that the chemotactic ability of PD-L1^+^ neutrophils is comparable to that of PD-L1^-^ neutrophils, suggesting that PD-L1 expression does not affect neutrophil chemotaxis.

To assess the pathogen-killing ability of neutrophils, we evaluated the ROS generation capacity of PD-L1^+^ neutrophils. ROS are highly reactive molecules derived from oxygen metabolism that play a crucial role in immune defense (24). In neutrophils, ROS production is one of the most well-known mechanisms for pathogen clearance, as it contributes to the “respiratory burst”, directly killing microbes and enhancing antimicrobial responses (24, 25). Fluorescent probe detection revealed that PD-L1^+^ neutrophils produced significantly lower levels of ROS compared to PD-L1^-^ neutrophils (*P*<0.001) (**Figures 4C, D**). When neutrophils reach the site of invasion, they bind to invading bacteria and eliminate them through phagocytosis (26, 27). We used fluorescently labeled E. coli bioparticles to assess the phagocytic activity of neutrophils (**Figures 4E, F**), and the results indicated that the phagocytic ability of PD-L1^+^ cells was significantly reduced compared with that of PD-L1^-^ cells (*P*<0.05). Degranulation is a key process by which neutrophils release antimicrobial and proinflammatory molecules (28). CD35 is a major biomarker of secretory vesicles, and CD63 is a marker of azurophilic granules. Granule exocytosis alters the composition of neutrophil surface proteins. Upon activation and enhanced degranulation, granule membranes fuse with the cytoplasmic membrane, leading to an increased expression of CD35 and CD63 on the cell surface (29). When detecting the fluorescence signals of CD35 and CD63 (**Figure 4G**), we observed a decrease in CD35 expression in PD-L1^+^ cells compared to PD-L1^-^ cells. These results demonstrate the reduced pathogen clearance capacity of PD-L1^+^ neutrophils, suggesting that the function of the PD-L1^+^ neutrophil subset is not primarily directed toward direct pathogen elimination and inflammation induction.

Neutrophils typically have a short lifespan, and apoptosis is the primary mode of cell death (30). After completing their antimicrobial functions, neutrophils undergo apoptosis through intrinsic (mitochondrial) or extrinsic (death receptor) signaling pathways and are subsequently cleared by phagocytes, such as macrophages (31). Flow cytometry analysis showed that the apoptosis level of PD-L1^+^ neutrophils was significantly lower than that of PD-L1^-^ cells (*P* < 0.01) (**Figures 4H, I**). This suggests that although neutrophils typically have a short lifespan, the PD-L1^+^ neutrophils may exhibit prolonged survival within the local immune microenvironment.

### 3.5 PD-L1^+^ neutrophils induce CD8^+^ T cell apoptosis and suppress CD8^+^ T cell polyfunctionality

**Figure 5:**
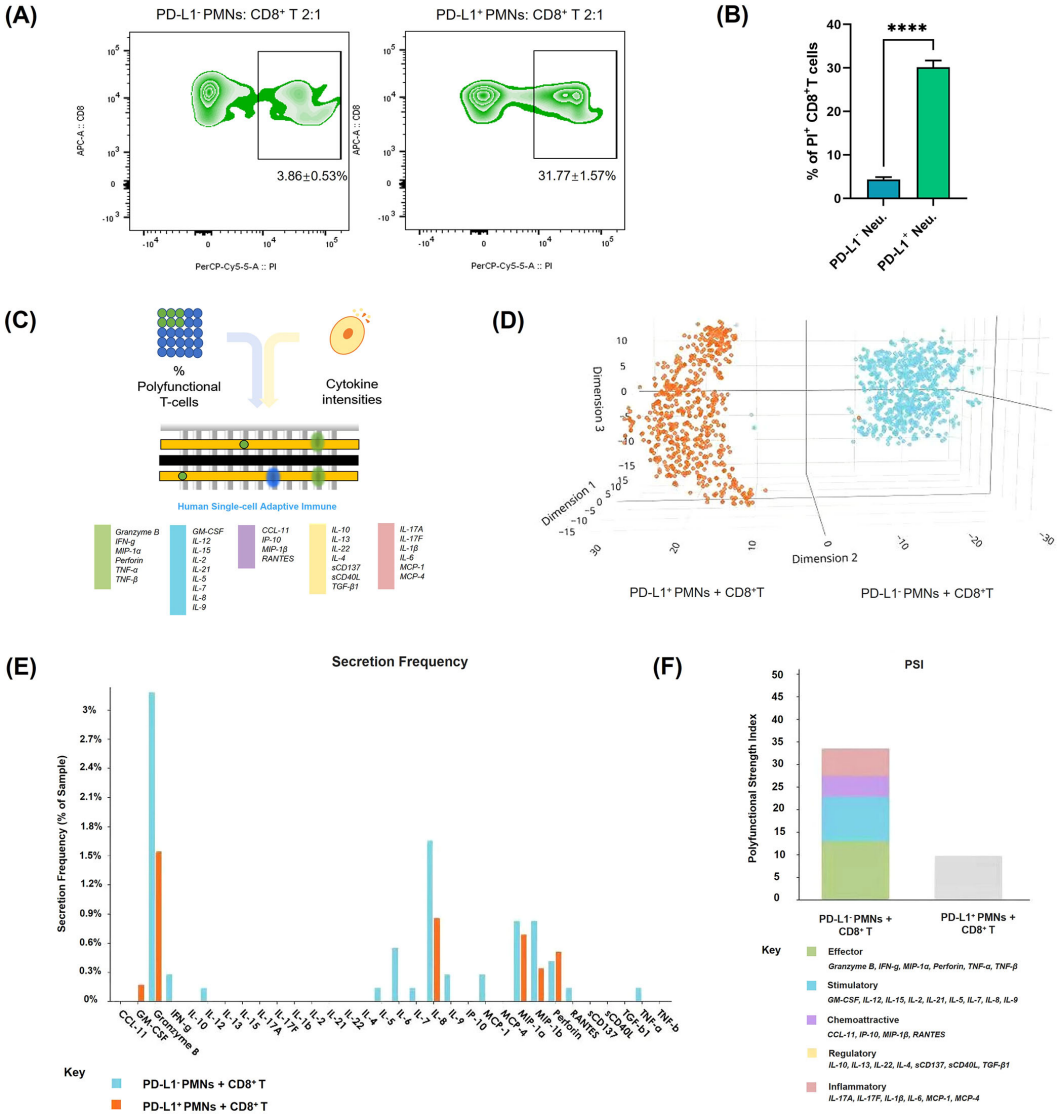
Apoptosis and functional changes of CD8^+^ T cells after co-culture with PD-L1^+^ neutrophils. **(A)** CD8^+^ T cell apoptosis was evaluated by flow cytometry after co-culturing with PD-L1^+^ or PD-L1^-^ neutrophils. The images are representative. **(B)** Quantification of the rate of PI-positive normalized to control CD8^+^ T cells (n = 3). **(C–F)** Analysis of CD8^+^ T cell polyfunctionality after co-culturing with PD-L1^+^ or PD-L1^-^ neutrophils (n = 3). **(C)** Schematic diagram of the single-cell IsoCode chip used for polyfunctionality analysis (18). **(D)** t-SNE plot showing dimensionality reduction of cytokine secretion profiles in CD8^+^ T cells under different conditions. **(E)** Frequency comparison of cytokine secretion by CD8^+^ T cells under different conditions. **(F)** Comparison of the polyfunctional strength index of CD8^+^ T cells under different conditions. Bar colors represent different cytokine components. ****P < .0001. T-SNE, t-distributed Stochastic Neighbor Embedding.

To investigate the impact of PD-L1^+^ neutrophils on CD8^+^ T cell survival and function, we co-cultured PD-L1^+^ and PD-L1^-^ neutrophils with autologous CD8^+^ T cells and assessed CD8^+^ T cell apoptosis and cytokine secretion profiles. Flow cytometry analysis demonstrated a significant increase in CD8^+^ T cell apoptosis following co-culture with PD-L1^+^ neutrophils compared to PD-L1^-^ neutrophils (*P* < 0.001) (**Figures 5A, B**; **Supplementary Figure 2A**). Moreover, the extent of CD8^+^ T cell apoptosis correlated with the proportion of PD-L1^+^ neutrophils in the co-culture, showing a progressive increase as the PD-L1^+^ neutrophil ratio increased (**Supplementary Figure 2B**).

**Figure 6:**
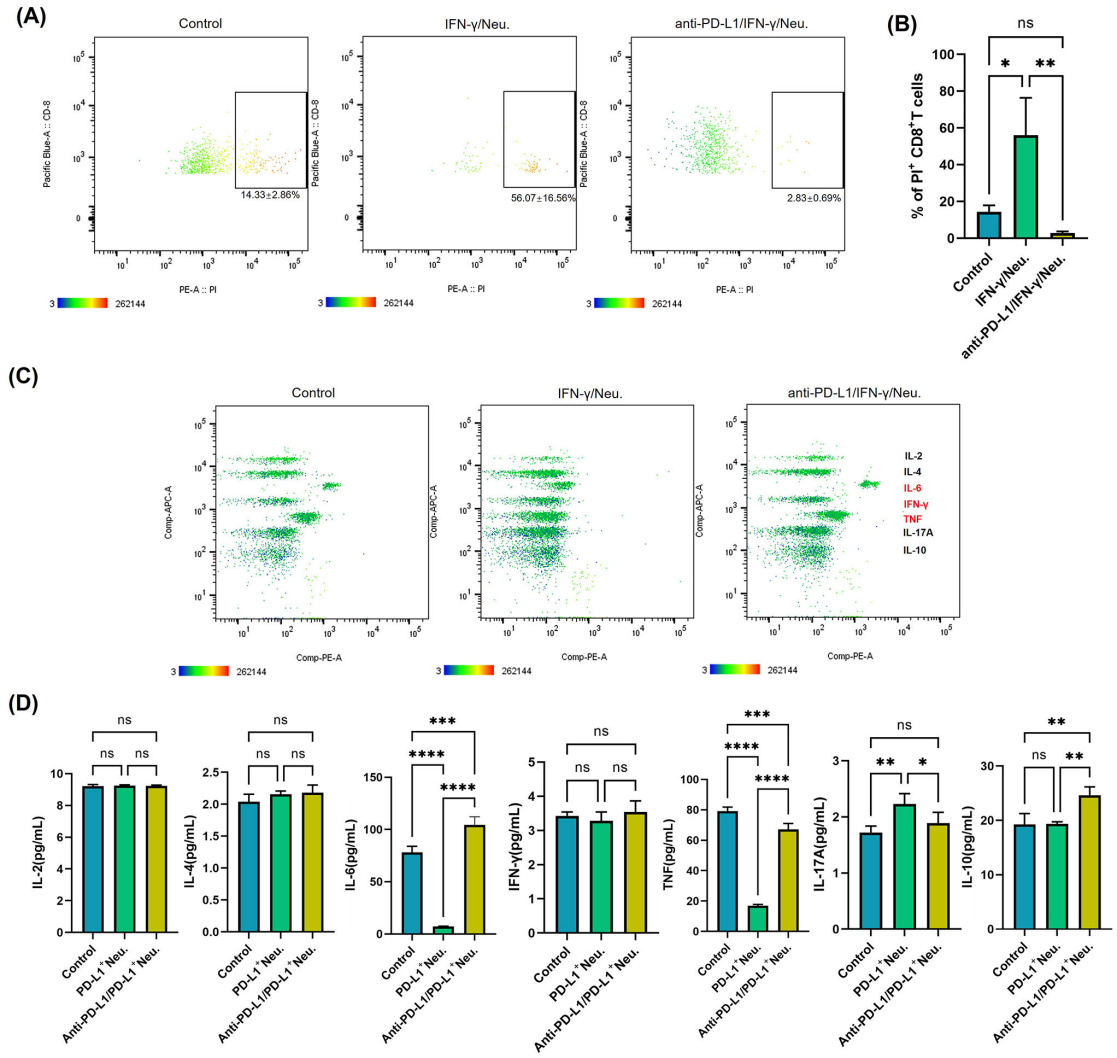
Targeting PD-L1 reverses CD8^+^ T cell apoptosis and functional suppression mediated by IFN-γ-induced PD-L1^+^ neutrophils. **(A)** Apoptosis analysis of CD8^+^ T cells after co-culturing with IFN-γ-treated neutrophils or PD-L1 monoclonal antibody (mAb) + IFN-γ-treated neutrophils. The images are representative. **(B)** Quantification of the rate of PI-positive CD8^+^ T cells in different groups normalized to control (n = 3). **(C)** Quantitative analysis of cytokine secretion from sorted CD8^+^ T cells after co-culturing with IFN-γ-treated neutrophils or PD-L1 mAb + IFN-γ-treated neutrophils, using the CBA kit. Representative images are shown. **(D)** Quantification of cytokine expression levels in CD8^+^ T cells after co-culturing with the above groups (n = 3). *P <.05, **P <.01, ***P <.001, ****P <.0001. ns, not significant. PI, propidium iodide; CBA, Cytometric Bead Array.

Additionally, to assess the functional consequences of CD8^+^ T cells after their interaction with PD-L1^+^ neutrophils, we performed polyfunctional single-cell cytokine analysis using an IsoCode chip-based platform (**Figure 5C**). t-SNE dimensionality reduction analysis (**Figure 5D**) of single-cell cytokine secretion indicated that PD-L1^+^ neutrophils significantly altered the cytokine expression profile of CD8^+^ T cells. The secretion frequency of various cytokines was significantly decreased after co-culture with PD-L1^+^ neutrophils, as evidenced by the markedly lower percentages of CD8^+^ T cells secreting granzyme B, IFN-γ, and TNF-α compared to those co-cultured with PD-L1^-^ neutrophils (**Figure 5E**). The polyfunctional strength index of CD8^+^ T cells was significantly reduced when co-culture with PD-L1^+^ neutrophils compared to PD-L1^-^ neutrophils, showing reduced expression of effector molecules, stimulatory factors, chemoattractive factors, regulatory factors, and inflammatory cytokines (**Figure 5F**). These findings indicate that PD-L1^+^ neutrophils inhibit CD8^+^ T cell activation. To further investigate the key role of PD-L1 in this process, we treated neutrophils with an anti-PD-L1 antibody (Atezolizumab, 10 µg/mL) in the presence of IFN-γ (10 ng/mL) prior to co-culture with CD8^+^ T cells. Flow cytometry analysis showed that PD-L1 blockade significantly reduced CD8^+^ T cell apoptosis compared to the group with IFN-γ-stimulated PD-L1^+^ neutrophils (*P* < 0.01) (**Figures 6A, B**). Cytokine secretion by CD8^+^ T cells was assessed using CBA kit. The results showed that, compared to co-culture with neutrophils induced to express PD-L1, PD-L1 blockade led to a significant increase in the secretion of the effector cytotoxic factor TNF and the pro-inflammatory cytokine IL-6 by CD8^+^ T cells (**Figures 6C, D**). These findings indicate that PD-L1^+^ neutrophils promote CD8^+^ T cell apoptosis and suppress their function through a PD-L1-dependent mechanism, whereas PD-L1 blockade effectively reverses this inhibitory effect.

## 4 Discussion

Halo nevus, also known as *leukoderma acquisitum centrifugum*, *Sutton nevus*, or perinevoid vitiligo (1, 3, 32), is a relatively common condition observed across different age groups, including both elderly individuals and children, with no significant sex-related differences in incidence (1, 2). The pathogenesis of halo nevi is currently believed to involve an immune response induced by mononuclear cell infiltration within and around the nevus, as well as the presence of antibodies targeting nevus cell antigens (4, 6, 7, 12). The regression of the central nevus and the appearance of the surrounding leukoderma are generally associated with the destruction of nevus melanocytes by peripheral T cells, as activated CD8^+^ T cells predominate in halo nevi (6, 11, 15). On the other hand, occasional neutrophil infiltration in halo nevi has been reported (11), while their roles within the local immune microenvironment remain insufficiently characterized. The findings of this study are consistent with previous research, revealing the presence of substantial numbers of CD8^+^ T cells and some neutrophils in both the central nevus and the surrounding leukoderma of halo nevi, accompanied by high expression of IFN-γ (**Figure 1**).

IFN-γ, which is highly expressed in halo nevi, is primarily secreted by activated CD8^+^ T cells (15). It exerts immunoregulatory effects and influences cell proliferation by binding to specific receptors on target cells and inducing the expression of a broad range of downstream genes (15, 33, 34). Among these, IFN-γ is one of the key regulators of the expression of PD-L1, a cell surface-expressed protein and a classical immune checkpoint molecule (21). Previous studies have shown that various cell types, including basophils, macrophages, neutrophils, and different cancer cells, can upregulate PD-L1 expression in response to IFN-γ stimulation (16). To further explore this relationship, we simulated the IFN-γ-rich environment observed in halo nevi and confirmed that high levels of IFN-γ can induce PD-L1 expression in neutrophils *in vitro* (**Figure 2**). We subsequently demonstrated the presence of PD-L1^+^ neutrophils in lesional tissues from halo nevus patients (**Figure 3**), consistent with the elevated local IFN-γ levels characteristic of this condition. In the GO analysis, we observed the enrichment of multiple membrane-related terms, which may be associated with the increased synthesis of membrane-localized PD-L1 following IFN-γ stimulation. Studies have shown that in certain diseases, IFN-γ can promote neutrophils to express PD-L1, contributing to a regulatory role in the disease. In the lungs of mice infected with *Mycobacterium tuberculosis*, neutrophils with low PD-L1 expression generate inflammasomes that respond to pathogenic mycobacteria, “accelerating” harmful inflammation. On the other hand, PD-L1 high-expressing neutrophils “control” the inflammatory response by inhibiting T lymphocyte proliferation and IFN-γ production. This beneficial PD-L1-dependent regulation is controlled by the IFN-γ receptor signaling pathway in neutrophils (35). Recent studies have found that during viral pneumonia, PD-L1^+^ neutrophils are remotely generated in the bone marrow in an IFN-γ-dependent manner and then migrate to the site of inflammation. Neutrophil depletion and PD-L1 blockade increase the susceptibility to experimental viral pneumonia. The study highlights the IFN-γ/PD-L1^+^ neutrophil axis as a feedback loop that regulates inflammation during severe viral pneumonia (36). Additionally, Huang et al. found that in sepsis, IFN-γ secreted by splenic infiltrating T lymphocytes can induce the generation of PD-L1^+^ neutrophils through the JAK2/STAT1 pathway (37). Although the JAK2/STAT1 pathway was not found to be enriched in our *in vitro* IFN-γ-induced neutrophil experiment, the MAPK pathway was enriched. Previous studies have confirmed that the MAPK/ERK pathway plays a key role in the IFN-γ-induced expression of PD-L1 in cells (23).

As key components of the innate immune system, neutrophils primarily participate in chemotaxis, pathogen phagocytosis, and release of ROS and inflammatory mediators (38, 39). Beyond directly eliminating pathogens, neutrophils can modulate adaptive immune responses through immune regulation (40–42). The PD-L1^+^ neutrophils exhibit unique functional characteristics (**Figure 4**). ROS production and phagocytosis are critical mechanisms through which neutrophils eliminate pathogens (43, 44). The reduced phagocytic capacity and ROS generation in the PD-L1^+^ neutrophil subset may indicate a shift toward an immunoregulatory role rather than direct pathogen clearance. Degranulation is not only one of the core antimicrobial mechanisms of neutrophils but is also a critical process for initiating and amplifying local inflammation (28). The reduced degranulation capacity of PD-L1^+^ neutrophils indicates a lower pro-inflammatory potential, which is consistent with the fact that halo nevi are not considered classical inflammatory diseases.

**Figure 7:**
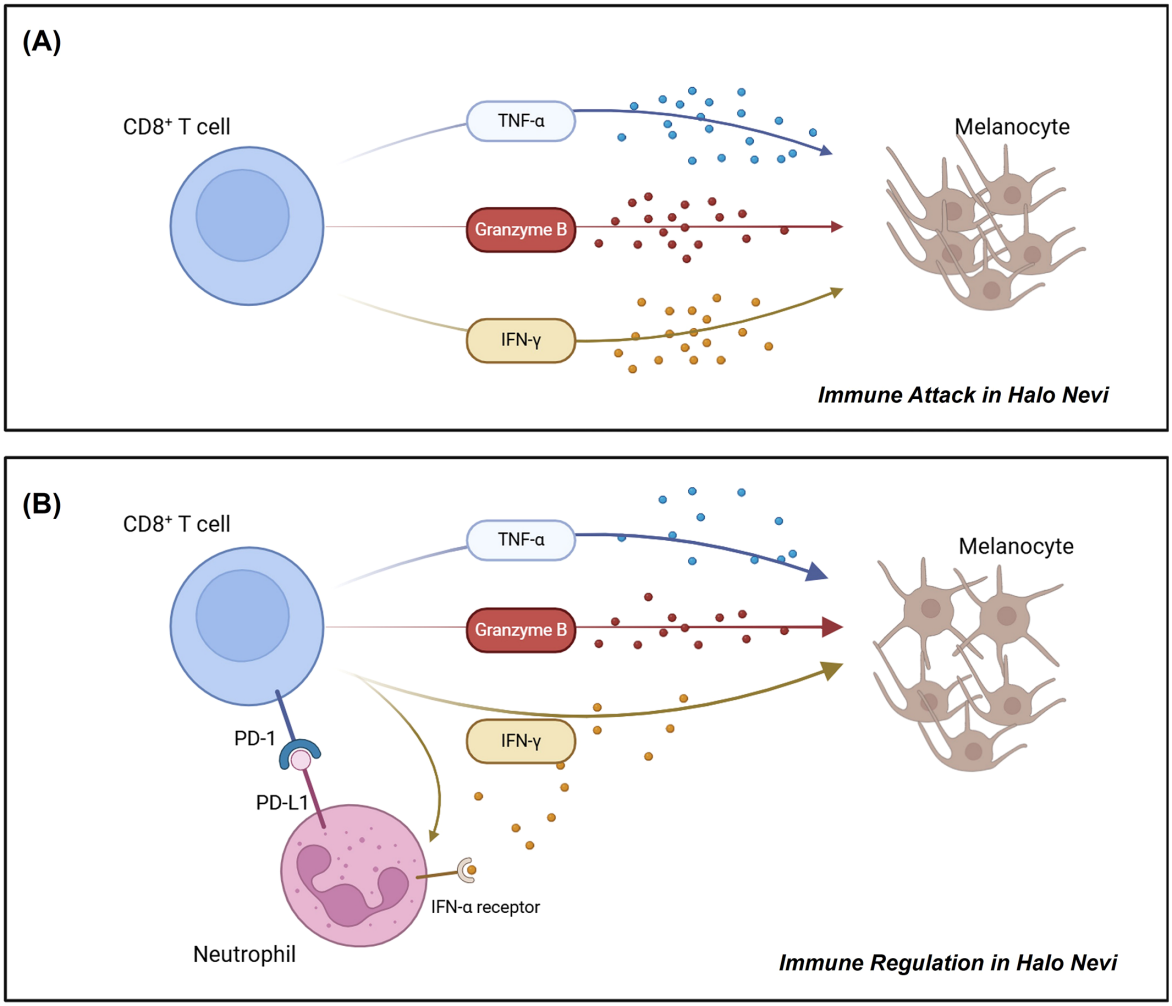
Schematic diagram illustrating the roles of CD8^+^ T cells and PD-L1^+^ neutrophils in halo nevi. **(A)** CD8^+^ T cells in halo nevi target and destroy melanocytes. **(B)** Neutrophils can acquire a PD-L1^+^ phenotype under IFN-γ stimulation, enabling them to suppress CD8^+^ T cell function and mitigate melanocyte damage.

PD-L1 can bind to PD-1 on T cells, exerting immunosuppressive effects in autoimmune regulation, tumor microenvironment, and chronic infections (45–47). While IFN-γ-induced PD-L1 expression on neutrophils has been reported to inhibit lymphocyte proliferation and promote apoptosis (48), there is limited research specifically addressing the capacity of PD-L1^+^ neutrophils to suppress T cell function. As mentioned earlier, functional analysis of PD-L1^+^ neutrophils suggests their close involvement in immune regulation; moreover, their spatial proximity to CD8^+^ T cells in the halo nevus further indicates a potential interaction between the two cell types (**Figure 3**). As expected, PD-L1^+^ neutrophils were found to not only promote CD8^+^ T cell apoptosis but also suppress their multifunctional cytokine secretion (**Figure 5**), whereas blockade of the PD-1/PD-L1 axis reversed these effects, confirming that neutrophils exert immunosuppressive functions on CD8^+^ T cells via PD-L1 (**Figure 6**). Moreover, the reduced apoptosis of PD-L1^+^ neutrophils (**Figure 4**) may contribute to a sustained immunosuppressive microenvironment. As PD-L1^+^ neutrophils can inhibit CD8^+^ T cell activity, their prolonged survival might enhance their capacity to regulate local immune responses and relieve cytotoxic damage to melanocytes (**Figure 7**).

Spontaneous lesion regression has been observed in some patients with halo nevi, typically beginning with the disappearance of the central pigmented nevus, followed by gradual repigmentation of the surrounding leukoderma (1). While regression of the nevus and the development of leukoderma are known to be mediated by CD8^+^ T cell infiltration (11), the mechanisms underlying the repigmentation of the surrounding area remain unclear. Based on our findings, we propose that PD-L1^+^ neutrophils may contribute to the regression of leukoderma, reflecting an endogenous immunoregulatory mechanism that helps restrain the progression of autoimmunity. In our study, CD66B^+^ neutrophil infiltration was observed in the lesional tissues of four out of six halo nevus patients (**Figure 1**). In the remaining two patients, immunofluorescence staining did not reveal significant CD66B expression. This finding is consistent with previous studies reporting occasional neutrophil infiltration in halo nevi (11, 49). Unlike classical inflammatory skin disorders, halo nevi are not typically characterized by robust neutrophilic infiltration, and the presence of neutrophils may vary between individuals. Potential confounding factors, such as the clinical progression stage of halo nevi, comorbid conditions (e.g., autoimmune or infectious diseases), and prior treatments (e.g., topical corticosteroids or immunomodulatory agents), may influence neutrophil infiltration and function, thereby contributing to the observed heterogeneity in neutrophil presence and activity among patients. Given the diverse clinical manifestations of halo nevi, with not all patients exhibiting a self-limiting disease course, our findings suggest that PD-L1^+^ neutrophil infiltration may predispose patients to regression of leukoderma.

Halo nevi and vitiligo are closely related, having similar pathogenic mechanisms (7, 50) and comparable immunological characteristics in terms of CD8^+^ T cell infiltration, high IFN-γ expression, and melanocyte loss (3, 7, 50). This study suggests that PD-L1^+^ neutrophils in halo nevi may play an important role in regulating CD8^+^ T cell activity, supporting the speculation that a comparable mechanism may underlie vitiligo as well. Vitiligo and halo nevi are generally not classified as classical inflammatory disorders; therefore, a small number of PD-L1^+^ neutrophils may not be sufficient to fully reverse disease progression. Future studies should explore whether targeting PD-L1^+^ neutrophils could serve as a potential therapeutic strategy for halo nevi and vitiligo. For instance, regulating PD-L1 expression may help modulate the immune microenvironment, thereby reducing CD8^+^ T cell-mediated melanocyte destruction.

This study has several limitations. The patient sample size was limited, as the study was based on lesional tissue samples from six patients with halo nevi, and neutrophil infiltration was observed in only four of them. Future large-scale clinical studies are needed to validate the relationship between neutrophils and disease prognosis. Moreover, this study has not investigated the recruitment mechanisms of PD-L1^+^ neutrophils in the disease, and future studies could combine single-cell sequencing or spatial transcriptomics to elucidate their origin and regulatory network further. In addition, there were differences between *in vitro* experiments and the *in vivo* environment. While *in vitro* PD-L1 inhibitors can reverse the immunosuppressive effect of PD-L1^+^ neutrophils on CD8^+^ T cells, whether this fully reflects the *in vivo* microenvironment still requires further validation. Given the challenges in establishing halo nevi-specific animal models, future studies should explore the use of vitiligo models to evaluate the therapeutic potential of PD-L1^+^ neutrophils.

In conclusion, this study provides new insights into the regulation of innate immunity in halo nevi. The halo nevus region exhibits a high expression of IFN-γ and the presence of neutrophils. IFN-γ induces PD-L1 expression on neutrophils, and these PD-L1^+^ neutrophils exert immunosuppressive effects on CD8^+^ T cells, which may help promote the self-limiting nature of the surrounding leukoderma. This research not only enhances our understanding of the self-regulatory mechanisms of halo nevi but also provides new perspectives for immune intervention in autoimmune diseases, such as vitiligo.

## Data Availability

Datasets related to this article can be found in the NCBI Gene Expression Omnibus (GEO) with accession number GSE295344.
