# Supplementary material for: PD-L1+ neutrophils mediate immune regulation of CD8+ T cells in halo nevi
Source: Front Immunol. 2025 Aug 20;16:1628913. doi: 10.3389/fimmu.2025.1628913 (PMC12405382; doi:10.3389/fimmu.2025.1628913)
Supplement: Supplementary file 1 [file DataSheet1.docx]

Supplementary Material

# Supplementary Figures


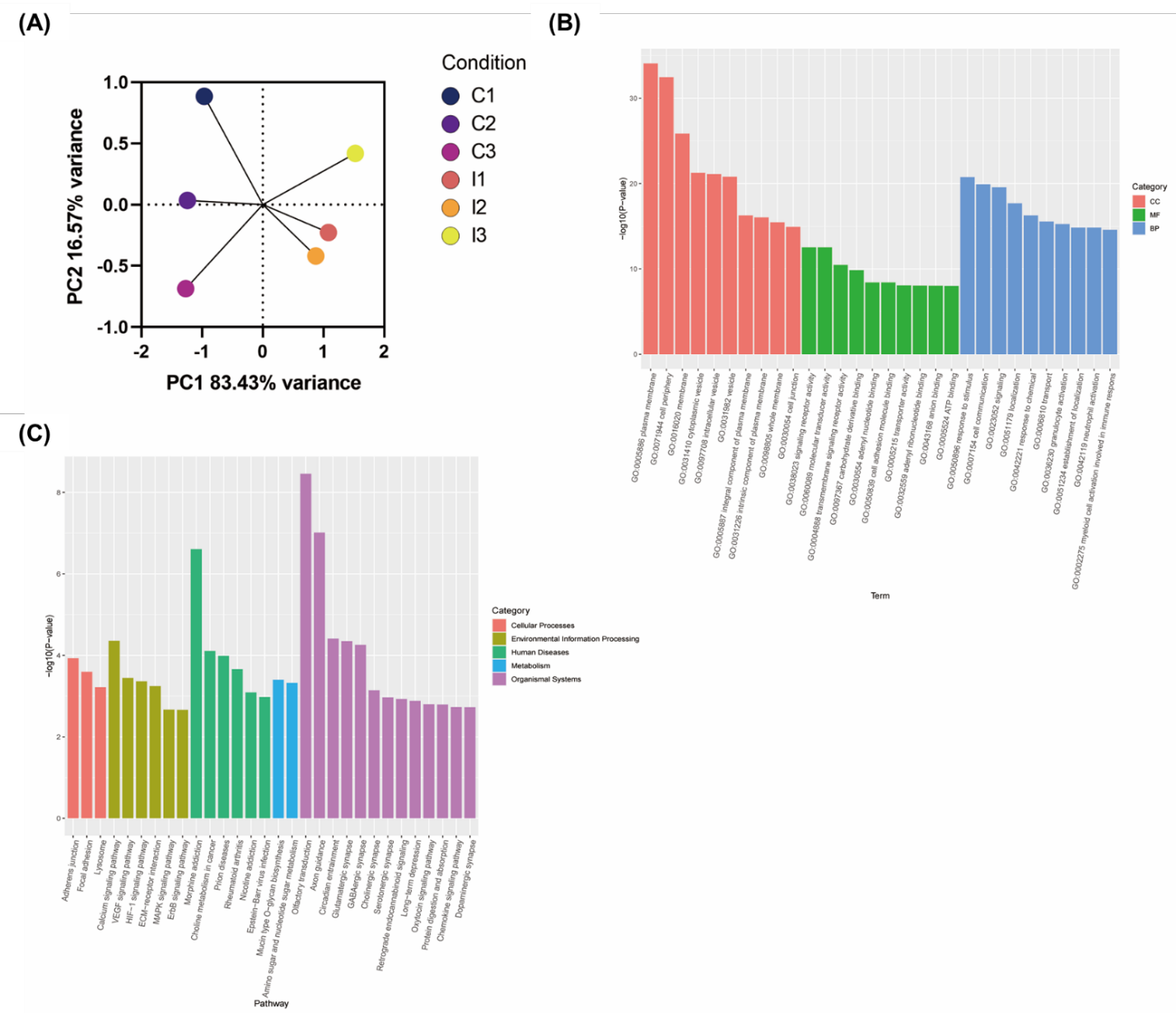


**Supplementary Figure 1.** **Transcriptomic characteristics and bioinformatic analysis of IFN-γ-treated neutrophils.** (A) Principal Component Analysis (PCA) of neutrophils stimulated with IFN-γ (10 ng/mL) in vitro compared to control neutrophils. Each sample is represented as a colored dot, with the X-axis corresponding to the first principal component and the Y-axis to the second. (B) Top 10 enriched GO terms in the categories of CC, BP, and MF for differentially expressed genes in IFN-γ-stimulated neutrophils versus control. (C) Top 30 enriched KEGG pathways for differentially expressed genes in IFN-γ-stimulated neutrophils versus control. GO, Gene Ontology; CC, Cellular Component; BP, Biological Process; MF, Molecular Function; KEGG, Kyoto Encyclopedia of Genes and Genomes.


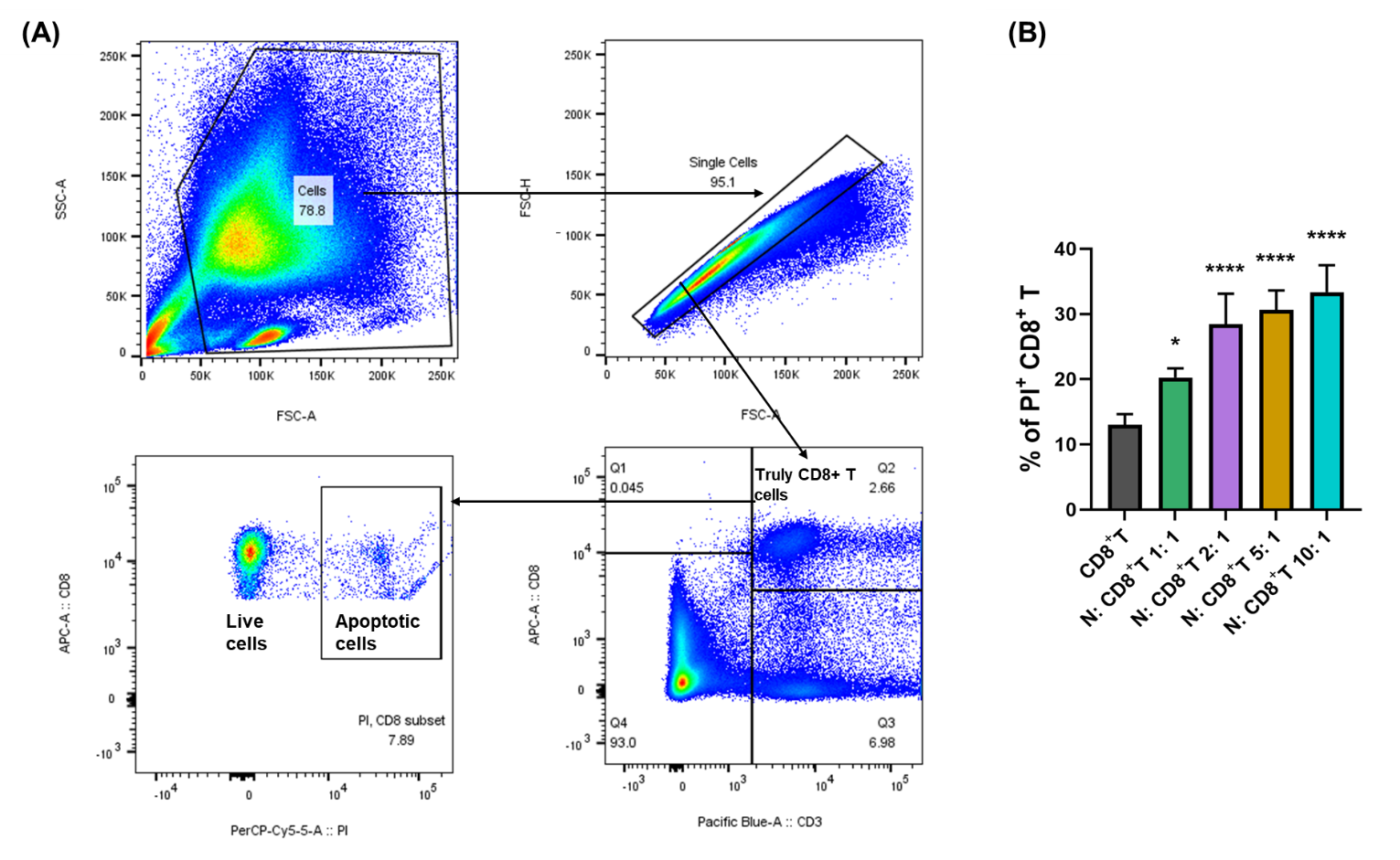


**Supplementary Figure 2. PD-L1⁺ neutrophils promote apoptosis following co-incubation with CD8⁺ T cells. (A) Gating strategy for CD8^+^ T cell apoptosis when neutrophils and CD8^+^ T cells are co-incubated by flow cytometry. The image is representative. (B) Apoptosis of CD8⁺ T cells when co-culturing with PD-L1⁺ neutrophils at different ratios (n=3). **P*＜.05, *****P*＜.0001.**
